# Zika Virus Infection in Patient with No Known Risk Factors, Utah, USA, 2016

**DOI:** 10.3201/eid2308.170479

**Published:** 2017-08

**Authors:** Elisabeth R. Krow-Lucal, Shannon A. Novosad, Angela C. Dunn, Carolyn R. Brent, Harry M. Savage, Ary Faraji, Dallin Peterson, Andrew Dibbs, Brook Vietor, Kimberly Christensen, Janeen J. Laven, Marvin S. Godsey, Bryan Christensen, Brigette Beyer, Margaret M. Cortese, Nina C. Johnson, Amanda J. Panella, Brad J. Biggerstaff, Michael Rubin, Scott K. Fridkin, J. Erin Staples, Allyn K. Nakashima

**Affiliations:** Centers for Disease Control and Prevention, Atlanta, Georgia, USA (E.R. Krow-Lucal, S.A. Novosad, C.R. Brent, B. Christensen, M.M. Cortese, N.C. Johnson, S.K. Fridkin);; Centers for Disease Control and Prevention, Fort Collins, Colorado, USA (E.R. Krow-Lucal, H.M. Savage, J.J. Laven, M.S. Godsey Jr., A.J. Panella, B.J. Biggerstaff, J.E. Staples);; Utah Department of Health, Salt Lake City, Utah, USA (A.C. Dunn, D. Peterson, K. Christensen, A.K. Nakashima);; Salt Lake County Health Department, Salt Lake City (C.R. Brent, A. Dibbs);; Salt Lake City Mosquito Abatement District, Salt Lake City (A. Faraji);; University of Utah, Salt Lake City (B. Vietor, B. Beyer, M. Rubin)

**Keywords:** Zika virus disease, Zika virus, viruses, infection, transmission, symptomatic, exposure, healthcare, person-to-person transmission, local transmission, arbovirus, vector-borne infections, zoonoses, Utah, United States

## Abstract

In 2016, Zika virus disease developed in a man (patient A) who had no known risk factors beyond caring for a relative who died of this disease (index patient). We investigated the source of infection for patient A by surveying other family contacts, healthcare personnel, and community members, and testing samples for Zika virus. We identified 19 family contacts who had similar exposures to the index patient; 86 healthcare personnel had contact with the index patient, including 57 (66%) who had contact with body fluids. Of 218 community members interviewed, 28 (13%) reported signs/symptoms and 132 (61%) provided a sample. Except for patient A, no other persons tested had laboratory evidence of recent Zika virus infection. Of 5,875 mosquitoes collected, none were known vectors of Zika virus and all were negative for Zika virus. The mechanism of transmission to patient A remains unknown but was likely person-to-person contact with the index patient.

Zika virus is an emerging mosquitoborne flavivirus transmitted primarily through the bite of infected *Aedes* (*Stegomyia*) mosquitoes. Other modes of transmission, including intrauterine, perinatal, sexual, blood transfusions, and laboratory exposure, have been described (*1**–**6*).

In June 2016, a 73-year-old man (index patient) died in a hospital in Salt Lake City, Utah, USA (*7*) (Figure 1). He had returned from Mexico 11 days previously and began feeling ill 3 days after his arrival in the United States. He sought care 2 days after illness onset and was hospitalized 3 days later. After admission, his health rapidly declined, and he died 3 days later of suspected dengue hemorrhagic shock syndrome. Postmortem testing identified Zika virus RNA in a blood sample obtained during hospitalization; the level of viremia in his serum sample was uncharacteristically high (*7**,**8*).

**Figure 1:**
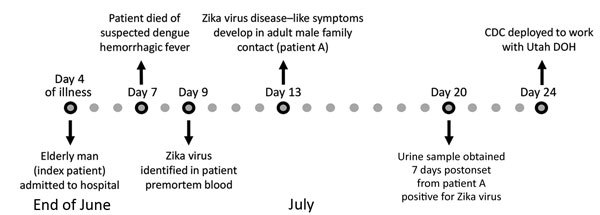
Timeline of events for investigation of Zika virus infection in patient with no known risk factors, Utah, USA, 2016. CDC, Centers for Disease Control and Prevention; DOH, Department of Health.

Six days after the death of the index patient, subjective fever, rash, and conjunctivitis developed in a 38-year-old man who was a family contact (patient A) (*7*). Patient A had not traveled to an area with ongoing Zika virus transmission, had not had sexual contact with a person who recently traveled to such an area, and had not received a blood transfusion or organ transplant. However, patient A had contact with the index patient during his period of viremia. Patient A also visited the 2 residences of the index patient after his death, suggesting possible vectorborne transmission from *Ae. aegypti* mosquitoes, which have been previously identified in Utah (*9*). Urine obtained from patient A 7 days after illness onset was positive for Zika virus RNA, and a day 11 serum sample was positive for Zika virus IgM and Zika virus and dengue virus neutralizing antibodies (*8*). Given the lack of travel or other risk factors for acquiring Zika virus for patient A, a public health investigation was launched to better define his exposures and determine a probable source of infection.

## Methods

For this investigation, we defined a contact as a person who resided in the same household with the index patient or who had direct contact with the index patient or his blood or other body fluids, such as conjunctival discharge, respiratory secretions, vomit, stool, or urine, when he was potentially viremic (defined as the date the index patient returned to the United States until his death). Evidence of recent infection was defined as a person with Zika virus RNA in serum or urine or Zika virus IgM and neutralizing antibodies in serum samples that were negative for neutralizing antibodies against dengue virus (*10*). We obtained consent for all persons who provided a sample or participated.

### Assessment of Person-to-Person Transmission

#### Evaluation of Family Contacts and Mortuary Workers

State and local health department staff interviewed all family members and friends identified as potential contacts of the index patient from the date when he was found to be positive for Zika virus through when patient A showed a positive test result. Interviewers asked about exposures to the index patient and any recent travel or vaccination that might affect Zika virus test results. All contacts were asked to provide a blood or urine sample to test for recent Zika virus infection. All community funeral and mortuary workers who had contact with the body of the index patient met the definition of a contact and were asked about their exposures and to provide a blood sample.

#### Assessment of Healthcare Personnel

Hospital medical records of the index patient were reviewed to characterize and quantify clinical conditions and procedures that generated blood or body fluid. A list of all healthcare personnel who potentially interacted with the index patient was generated on the basis of employee assignments. Healthcare personnel with possible contact were called and an interview was scheduled. If after 2 attempts they could not be reached, they were categorized as not reachable. Healthcare personnel were interviewed to determine the level of interaction (e.g., type of contact, type of care provided, exposure to blood or body fluids, use of personal protective equipment [PPE]); recent travel; and vaccinations. Healthcare personnel were defined as having other concerning factors if they reported being pregnant, were attempting to become pregnant, or had >2 signs/symptoms consistent with Zika virus infection (i.e., fever, rash, arthralgia, or conjunctivitis) in the previous 30 days. All employees with direct contact or other concerning factors were asked to provide blood or urine samples for Zika virus testing (*11*).

### Assessment of Potential Vectorborne Transmission

#### Vector Assessment

Local mosquito abatement districts worked in collaboration with the Centers for Disease Control (CDC, Fort Collins, CO, USA) to conduct larval and adult mosquito surveillance in the 3 areas where the index patient (2 residences) and patient A resided. Door-to-door household surveys were conducted, and light traps, CO_2_ traps, gravid traps, and BioGents traps (BioGents, Regensberg, Germany) were deployed to collect different species at sites around residences of patient A and the index patient. During mid-July, mosquito abatement district adult mosquito collection was performed for 2 days. Five days after the first trapping, CDC and local mosquito abatement districts collected mosquitoes at 12 sites in each of the 3 areas described. Specifically, 18 BioGents-2, 6 gravid, and 6 CDC light traps with CO_2_ were used for 30 traps/day. Ovicups were set at 9 sites to detect container-inhabiting *Aedes* mosquitoes. Adult mosquitoes were shipped on dry ice to CDC in Fort Collins for processing and testing.

Potential larval and pupal habitats were inspected at the 3 residences of interest and nearby homes. Aquatic stages were collected and transported to mosquito control district facilities for rearing and identification.

#### Community Assessment

All households within a 200-m radius of the 2 properties where the index patient stayed while potentially viremic were surveyed. We determined this radius on the basis of a compromise between the likely movement of *Aedes* mosquitos (estimated as ≈30–450 m/d) and the number of households that could be surveyed (*12*). A household was eligible for inclusion if >1 resident of the house had resided in the household for the month before illness onset of patient A. A person who slept in the house >2 days/week was considered a household member.

Teams visited households in late July for 4 days, provided information about the investigation, and obtained verbal consent. If the person did not wish to participate, they were considered refusing. If no residents or heads of household were available at the initial visit, they were revisited 2 more times at a different time and day. If after 3 visits no one answered, the household was considered not available. For participating households, verbal consent or assent to participate was obtained from all household members >12 years of age. For children <18 years of age, permission was obtained from parents or legal guardians.

We surveyed all consenting household members by using a questionnaire that captured information on demographics, signs/symptoms of possible Zika virus infection, recent travel or sexual contact with a traveler, receipt of flavivirus vaccines, pregnancy status, exposures to mosquitoes, and personal and household protective measures. We defined signs/symptoms of possible Zika virus infection as fever, rash, conjunctivitis, or arthralgia with onset after the date of return of the index patient to the United States. Persons whose signs/symptoms began before the return of the index patient or were explained by an alternate etiology (e.g., culture-proven bacterial infection) were not included among those with reported signs/symptoms. After completion of the survey, we asked each household member >6 months of age to provide a blood sample; for participants symptomatic within the previous 2 weeks, a urine sample was also collected. We did not collect Samples from infants <6 months of age because interpretation of results would be complicated by maternally derived antibodies. However, parents did respond to the questionnaire for those infants.

### Laboratory Testing

Serum samples were tested by using a Zika virus IgM capture ELISA at CDC (Fort Collins) or the Utah Public Health Laboratory (Salt Lake City, UT, USA) per standard protocol (*13**,**14*). Samples positive for Zika virus IgM were confirmed by using a 90% plaque reduction neutralization test at CDC (Fort Collins) (*15*). Urine samples were tested by using a reverse transcription PCR (RT-PCR) (Trioplex assay) for Zika virus at the Utah Public Health Laboratory (*14*). If a person reported signs/symptoms in the past week, then their serum sample was also tested by RT-PCR for Zika virus RNA per testing guidelines (*13*). Mosquito pools were tested for Zika virus and West Nile virus RNA by using described methods (*16*).

### Data Collection and Analysis

We entered survey data with unique identification numbers for participants into either REDCap (https://www.project-redcap.org/) or Epi Info (CDC) databases for analysis. We summarized continuous variables as medians and ranges and dichotomous variables as frequencies and proportions. For the community assessment, we first estimated the number of persons residing in the areas around residences of the index patient on the basis of average household size values at the ZIP code level obtained from the 2010 US Census (*17*). We then used the hypergeometric distribution to calculate the probability that a nonparticipant residing in the areas near the residences of the index patient could have been infected with Zika virus.

Procedures and data collection tools for healthcare personnel and community assessments were reviewed by human subject advisors at the Utah Department of Health and CDC. These procedures and tools were determined to be part of a nonresearch public health response.

## Results

### Evaluation of Family Contacts and Mortuary Worker

A total of 22 family members or friends potentially interacted with the index patient from his return to the United States until after his death; 19 (86%) met the definition of a contact. Of the 19 family contacts, 15 resided with or visited the index patient at his residences, and 13 visited him at the hospital. The most common interactions with the index patient included kissing, primarily on the cheek (n = 6), and assisting in care (e.g., cleaning up vomit, stool, or urine, or wiping tears) (n = 6). These activities were performed without PPE. Twelve community mortuary workers also interacted with the index patient in the hospital or mortuary, and all met the definition of a contact. Other than patient A, no other contact reported a Zika-like illness after their interaction with the index patient. 

Of the 19 family or friend contacts, 18 were negative for Zika virus IgM in serum (n = 14) or Zika virus RNA by PCR in urine (n = 17). Only patient A had recent evidence of Zika virus infection. All 12 mortuary workers were negative for Zika virus IgM in serum.

The most recent travel of patient A was to Mexico >1 year earlier. He had not had sexual contact with someone who had recently traveled to an area where Zika virus was known to be circulating and had not received any blood transfusions or organ transplants. He did not have any serious underlying conditions and was not immunosuppressed. Similar to other family members, patient A visited the residences where the index patient was staying before the index patient was hospitalized and after his death. Before hospitalization of the index patient, patient A had only casual contact (e.g., hugging and kissing) with the index patient.

During hospitalization of the index patient, patient A reported staying for 2 days and nights (>48 hours) in his room in the intensive care unit (ICU) and reported hugging, kissing, and touching him frequently. He assisted hospital staff in moving the index patient after a bowel movement, but did not come into contact with fecal matter or any other body fluid. Patient A had no breaks in his skin, including no chronic skin conditions, oral lesions, recent dental work, or needle exposures. Interactions of patient A with the index patient were similar to those reported by other family members. The wife of the index patient reported more frequent and direct contact (assisted with bodily functions, patient cleaning, and in-home care) with the index patient than patient A.

### Assessment of Healthcare Workers

**Figure 2:**
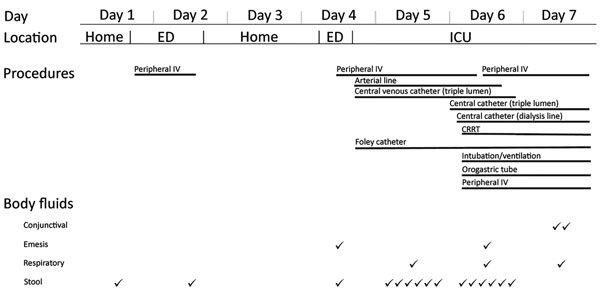
Characteristics of clinical course investigation of Zika virus infection in patient with no known risk factors, Utah, USA, 2016. Patient location, procedures, and body fluid output are as documented in medical records by day of treatment or observation of index patient. Body fluid output is separate from potential exposures generated by procedures. CRRT, continuous renal replacement therapy; ED, emergency department; ICU, intensive care unit; IV, intravenous line; ✓, recorded output (frequency).

The index patient was evaluated in the emergency department twice before being admitted and transferred to an ICU for the duration of his hospitalization. He required intensive clinical care, including mechanical ventilation, hemodialysis (continuous renal replacement therapy), and multiple procedures, including central and arterial line placement and endotracheal intubation (Figure 2). These procedures provided opportunities for contact of healthcare personnel with blood or other body fluids.

A total of 132 healthcare personnel were identified as having potential contact with the index patient, or his immediate environment, waste, or medical equipment. Twenty denied any interaction with the index patient and 14 were not reachable, resulting in 98 (74%) available for a complete interview. Of these personnel, 86 (88%) reported contact with the index patient or his immediate environment or had other concerning factors.

Of the 86 workers, 54 (63%) had contact with the index patient in the ICU and 26 (31%) had contact with the index patient in the emergency department. Most (72, 84%) workers provided direct patient care, and 57 (66%) reported contact with blood or other body fluids (Table 1). These 57 healthcare workers reported 128 separate exposures to blood or body fluids, including 39 (30%) exposures to blood, 35 (27%) to sweat, 18 (14%) to respiratory secretions, 15 (12%) to urine, 10 (8%) to stool, 8 (6%) to tears, and 3 (2%) to vomit. The most common PPE ensemble worn during these encounters was gloves only (81 workers, 63%) followed by gloves and gown (23, 18%); 10 (8%) encounters occurred without any PPE being used, including 8 encounters with sweat and 2 with tears (Table 1). No healthcare workers reported blood or body fluid contact with nonintact skin or mucous membranes, and there were no percutaneous exposures. Two healthcare workers reported having blood-soaked scrubs after postmortem cleaning of the body.

**Table 1:** Characteristics of 128 encounters by 57 healthcare personnel with index patient by type of PPE used during investigation of Zika virus infection in patient with no known risk factors, Utah, USA, 2016*

PPE	Blood, n = 39	Respiratory/GI/GU, n = 46	Sweat, n = 35	Conjunctival, n = 8	Total, n = 128
None	0 (0)	0 (0)	8 (23)	2 (25)	10 (8)
Gloves only	29 (74)	27 (59)	21 (60)	4 (50)	81 (63)
Gloves and gown	8 (21)	11 (24)	3 (9)	1 (13)	23 (18)
Other	2 (5)	6 (13)	0 (0)	0 (0)	8 (6)
Unknown	0 (0)	2 (4)	3 (9)	1 (13)	6 (5)

*Values are no. (%) persons. GI, gastrointestinal; GU, genitourinary; PPE, personal protective equipment.

Eighty (93%) of 86 healthcare workers provided blood samples for testing, and 1 person with recent signs (rash and conjunctivitis) provided a urine sample for testing. All 80 (100%) serum samples were negative for Zika virus IgM, and the 1 urine sample was negative for Zika virus by RT-PCR.

### Assessment of Vectorborne Transmission

#### Vector Surveillance

Larvae and pupae collected at the 3 residences and nearby areas included only *Culex* and *Culiseta* species (*Cx. pipiens*, *Cx. tarsalis*, *Cs. incidens*, and *Cs. inornata*). Inspection of egg papers from ovicups failed to detect viable mosquito eggs. Mosquito collections from all traps failed to detect the invasive species *Aedes aegypti* or *Ae. albopictus*. A total of 5,875 adult mosquitoes representing 7 species (4,765 *Cx. pipiens*, 658 *Cx. tarsalis*, 4 *Culex* spp., 299 *Cs. incidens*, 138 *Cs. inornata*, 7 *Ae. dorsalis*, 1 *Ae. vexans*, 2 *Aedes* spp*.,* and 1 *Anopheles freeborni*) were collected and tested for virus in 501 pools. All mosquito pools were negative for Zika virus by RT-PCR. However, 2 pools containing female *Cx. pipiens* mosquitoes were positive for West Nile virus RNA by RT-PCR with screening and confirmatory primers.

#### Community Survey

There were 226 occupied households within a 200-m radius of the residences where the index patient stayed. Of these households, 89 (39%) had >1 resident who completed a survey; 72 (32%) were not available, 62 (27%) refused participation, and 3 (1%) were excluded because residents were unable to be interviewed in their native language.

From the 89 participating households, 218 persons completed a questionnaire (for themselves or family members <12 years of age). Of the 218 participants, 119 (55%) were female; median age was 35 years (range 4 months–80 years) (Table 2). Most (150, 69%) participants spent an average of >1 hours/day outdoors during the preceding month. However, most (172, 79%) reported never wearing insect repellent. Less than half (87, 40%) reported being bitten by mosquitoes in the preceding month. Only 22 (10%) participants reported traveling in the preceding year, but most of them (20, 91%) reported traveling to a country where Zika virus was known to be circulating. Twenty-eight (13%) participants reported having >1 of the 4 Zika virus–associated signs/symptoms in the month before their interview; 15 (7%) reported having >2 signs/symptoms.

**Table 2:** Characteristics of 218 community survey questionnaire respondents during investigation of Zika virus infection in patient with no known risk factors, Utah, USA, 2016

Characteristic	No. (%)	
Demographic		
Age, y	
<20	65 (30)	
20–39	57 (26)	
40–59	53 (24)	
>60	31 (14)	
Unknown	12 (6)	
Sex		
F	119 (55)	
M	99 (45)	
Pregnant	2 (1)	
Born outside United States	63 (29)	
Exposures in 30 d before survey		
Average time outdoors/d, h		
<1	55 (25)	
1–4	108 (50)	
5–10	24 (11)	
>10	18 (8)	
Unknown	13 (6)	
Repellent use while outdoors		
Always	8 (4)	
Most of the time	3 (1)	
Sometimes	21 (10)	
Never	172 (79)	
Unknown	14 (6)	
Recent mosquito bites	87 (40)	
Screens on windows		
All	72 (33)	
Most	33 (15)	
Some	28 (13)	
None	13 (6)	
Never leave windows or doors open	52 (24)	
Unknown	20 (9)	
Travel out of country in previous year		
Traveled internationally	22 (10)	
Travel location		
South America	19 (9)	
Central America	1 (0)	
Caribbean	3 (1)	
Europe	1 (0)	
Asia	3 (1)	
Zika virus–like signs/symptoms in month before survey		
No. reported signs/symptoms		
0	181 (83)	
1	13 (6)	
2	14 (6)	
3	1 (<1)	
4	0 (0)	
Unknown	9 (4)	
Type of signs/symptoms reported		
Fever	16 (7)	
Rash	9 (4)	
Conjunctivitis	5 (2)	
Joint pain	14 (6)	

Of the 218 participants, 132 (61%) also provided *>*1 sample, including 124 who provided only a blood sample, 6 who provided blood and urine samples, and 2 who provided only a urine sample. Of the 130 blood samples, 2 were positive for Zika virus IgM. However, these results for the 2 samples were not confirmed by plaque reduction neutralization test (these samples were negative for Zika virus and dengue virus neutralizing antibodies). Because these 2 samples were obtained from asymptomatic persons, urine samples were not obtained. All other serum samples were negative for Zika virus IgM. All 8 urine samples were negative for Zika virus RNA.

Given the number of persons estimated to live in households in the survey area and who provided a specimen, and that no positive samples were observed, we found that the exact upper 95% confidence limit for the proportion of Zika virus–positive persons in the (untested) population of nearby residents was 2.0% when we used sampling without replacement and the finite population. Thus, it is highly likely that <2.0% of unsampled persons would have been infected with Zika virus.

## Discussion

Our investigation of patient A did not identify the probable source of his infection and did not identify any additional persons recently infected with Zika virus among family contacts, healthcare workers, or community members. The index patient was unique when compared with other persons with Zika virus disease because his illness was fatal and his relative viral load was estimated to be ≈100,000 times higher than the average level reported (*8*). These characteristics, combined with a lack of additional exposure for patient A, make it likely that the index patient was the source of infection for patient A, although inability to sequence virus obtained from patient A prevented a definitive confirmation (*7*). None of the other family members became infected, despite similar or more frequent and direct contact with the index patient during his viremic period. No healthcare personnel became infected despite the index patient having substantial invasive procedures and moderate production of body fluids. Overall, our findings suggest the infection of patient A represents a rare transmission event through unknown, but likely, person-to-person mechanisms.

On the basis of reported contact of patient A with the index patient, patient A might have been exposed to saliva or tears of the index patient, although patient A had no skin lesions or noted mucous membrane exposures that would have increased his likelihood of becoming infected, particularly when compared with other family members. To date, Zika virus has been detected by viral culture in several body fluids, including blood, urine, amniotic fluid, a conjunctival swab specimen, breast milk, semen, and saliva (*18**–**22*). Zika virus RNA also has been detected in cerebrospinal fluid, aqueous humor, cervical mucous, and nasopharyngeal, vaginal, and endocervical swab specimens (*23**–**26*). However, our knowledge about the timing and amount of Zika virus in blood or body fluids of the index patient was limited. Thus, we are unable to definitively state how patient A was infected.

Although the index patient had a high level of viremia, no healthcare personnel showed evidence of recent Zika virus infection. There were >100 reported encounters with blood and other body fluids with a variety of PPE reflecting standard precautions, which is probably representative of care given to patients in an ICU (*27*). This finding suggests that healthcare workers caring for severely ill patients with Zika virus disease should continue to use standard precautions with correct PPE when handling body fluids to prevent infection (*28*).

Although *Ae. aegypti* mosquitoes were previously identified in southwestern Utah in 2013 (*9*), our vector investigations did not identify *Ae. aegypti* or *Ae. albopictus* mosquitoes. None of the other mosquitoes collected were positive for Zika virus, but 2 pools were positive for West Nile virus, a finding that supports the efficacy of entomologic surveillance. In addition, no persons tested in the 200-m radius around the households in which the index patient stayed had evidence of a recent Zika virus infection. Although low-level transmission could not be definitively ruled out, results of the vector and community investigations do not support the suggestion that patient A was infected by a mosquito that had fed on the index patient before his hospitalization.

Our investigation had several limitations. First, although family contacts and healthcare workers were interviewed several times by professionals trained in interview techniques, recall bias regarding specific exposures they might have had with the index patient was likely. Second, we probably did not identify all healthcare workers who had contact with index patient because information was obtained retrospectively from the chart for the patient and staffing schedule. Third, documentation in the medical records regarding type and amounts of body fluids might have been incomplete, leading to underestimation of healthcare personnel exposure. Fourth, because of incomplete participation in the community survey, we might have missed persons who were infected by Zika virus in the community, an event we estimated to be low. These limitations might have prohibited identification of an alternate source of infection for patient A. We did not explore potential differences in susceptibility between patient A and other contacts of the index patient but focused on exposure. Thus, other factors, such as history of flavivirus infection in contacts of the index patient, might have contributed to a difference in susceptibility to infection between patient A and other persons.

Currently, Zika virus is known to be transmitted by the bite of an infected mosquito, congenitally from an infected mother to her fetus, sexually, through blood transfusion, and by laboratory exposure (*1*–*6*). Healthcare providers and public health officials should be aware that person-to-person transmission beyond sexual transmission might occur, albeit rarely, and should be investigated to determine the potential source of infection by obtaining various body fluids from persons suspected of transmitting the virus to another person through an undetermined route. Additional investigation is needed to determine the infectious risk various body fluids represent for person-to-person transmission and to determine host factors that might increase susceptibility for infection.
